# Transcriptomic analysis identifies CYP27A1 as a diagnostic marker for the prognosis and immunity in lung adenocarcinoma

**DOI:** 10.1186/s12865-023-00572-1

**Published:** 2023-10-10

**Authors:** Yi Yin, Muqun He, Yunjian Huang, Xianhe Xie

**Affiliations:** 1 Department of Medical Oncology, Clinical Oncology School of, Fujian Medical University, Fujian Cancer Hospital, Fuzhou, 350014 China; 2 Department of Oncology, Molecular Oncology Research Institute, Fujian Key Laboratory of Precision Medicine for Cancer, The First Affiliated Hospital of Fujian Medical University, Fuzhou, 350005 China

**Keywords:** Immune infiltrates, TCGA, Lipid metabolism, Lung cancer, Prognosis

## Abstract

**Background:**

The association between lipid metabolism disorder and carcinogenesis is well-established, but there is limited research on the connection between lipid metabolism-related genes (LRGs) and lung adenocarcinoma (LUAD). The objective of our research was to identify LRGs as the potential biomarkers for prognosis and assess their impact on immune cell infiltration in LUAD.

**Methods:**

We identified novel prognostic LRGs for LUAD patients via the bioinformatics analysis. CYP27A1 expression level was systematically evaluated via various databases, such as TCGA, UALCAN, and TIMER. Subsequently, LinkedOmics was utilized to perform the CYP27A1 co-expression network and GSEA. ssGSEA was conducted to assess the association between infiltration of immune cells and CYP27A1 expression. CYP27A1’s expression level was validated by qRT-PCR analysis.

**Results:**

CYP27A1 expression was decreased in LUAD. Reduced CYP27A1 expression was linked to unfavorable prognosis in LUAD. Univariate and multivariate analyses indicated that CYP27A1 was an independent prognostic biomarker for LUAD patients. GSEA results revealed a positive correlation between CYP27A1 expression and immune-related pathways. Furthermore, CYP27A1 expression was positively correlated with the infiltration levels of most immune cells.

**Conclusion:**

CYP27A1 is a potential biomarker for LUAD patients, and our findings provided a novel perspective to develop the prognostic marker for LUAD patients.

**Supplementary Information:**

The online version contains supplementary material available at 10.1186/s12865-023-00572-1.

## Introduction

Based on the GLOBOCAN 2020 estimates of cancer mortality and incidence, lung cancer continues to be a major contributor to cancer-related mortality and is the prevailing form of cancer worldwide [1]. Lung adenocarcinoma (LUAD) is the predominant subtype of lung cancer, representing approximately 40% of all cases [2]. Despite some progress in understanding the pathogenesis and developing novel therapies for LUAD, it continues to be one of the most lethal and aggressive forms of tumors, with an overall survival rate of less than five years [3]. In recent years, there has been significant focus on immunotherapy as a treatment for cancer. However, only 20–30% of patients received effective treatment [4]. Hence, it is imperative to discover new prognostic biomarkers to aid in the diagnosis and prevention of LUAD patients.

An increasing body of research has demonstrated the crucial involvement of aberrant metabolic reprogramming, such as mitochondrial oxidative phosphorylation [5], cholesterol metabolism [6], fatty acid metabolism [7], and glycolysis [8], in the initiation and progression of cancer. The development of tumors can lead to the modification of metabolic pathways, which in turn promote the survival and proliferation of tumor cells within the tumor microenvironment [9, 10]. Cholesterol and fatty acids are the basic structure of cell membranes, contributing to the metastasis, proliferation, and invasion of tumor cells [11]. Lipids play a significant role in transmitting signals within cancer cells and contribute to the energy supply needed for cancer development [12]. The occurrence of bladder cancer is related to the change in lipid metabolism [13]. Fatty acid synthase (FASN) is served as a prognostic marker of bladder cancer development [14]. Previous findings indicated that the involvement of factors associated with lipid metabolism can lead to a reduced incidence of cancer [15]. Furthermore, MYC expression contributes to abnormal lipid metabolism that is important for lung cell growth and proliferation [16]. These results indicated the crucial role of lipid metabolism in the development of tumors, indicating that lipid metabolism-related genes (LRGs) hold significant potential as the prognostic markers for patients with LUAD.

We retrieved the LUAD-reltaed dataset from the Cancer Genome Atlas (TCGA) database to identify prognosis-related LRGs. Among these LRGs, we focused on investigating the role of CYP27A1, a gene with unclear function in LUAD. We conducted prognosis analysis, gene set enrichment analysis (GSEA), and immune infiltration analyses to further examine the significance of CYP27A1 in LUAD.

## Methods

### Collection of dataset and patients data

The RNA-seq (FPKM) data and clinicopathological information of LUAD patients were downloaded from the TCGA database. The dataset consisted of 59 adjacent noncancer samples and 535 tumor samples. Furthermore, mRNA profiles obtained from Gene Expression Omnibus (GEO) including GSE41271, GSE11969, and GSE30219 were downloaded and utilized as validation datasets for lung cancer patients.

### Collection of LRGs

LRGs were obtained from the lipid metabolism-related data sets (Reactome phospholipid metabolism, Reactome metabolism of lipids, lipid raft, KEGG glycerophospholipid metabolism, and hallmark fatty acid metabolism) (Table S1).

### Identifying genes with differential expression (DEGs)

The limma package in R was utilized to identify the DEGs between the tumor samples and adjacent noncancer samples (p.adj < 0.05 and ∣logFC∣ ≥ 1) [17]. The volcano plots were generated using ggplot2.

### Identification of prognosis-related genes (PRGs)

The survival package was applied to screen PRGs from TCGA-LUAD based on a p-value < 0.05. The Venny tool was employed to identify the overlapping set of LRGs, DEGs, and PRGs. Subsequently, these differentially expressed prognosis-associated LRGs were visualized on a forest plot using the ggplot2 package.

### Analysis of CYP27A1 expression in LUAD using various databases

The differentially expressed prognosis-associated LRGs were uploaded to the STRING database (https://cn.string-db.org/) for building the protein–protein interaction (PPI) network. The result was visualized using Cytoscape software (version 3.2.1). The cytoHubba plugin of Cytoscape software was employed to identify the core gene (CYP27A1) based on its degree value. In TIMER database, we used the “DiffExp module” to assess the CYP27A1 expression in pan-cancer. The UALCAN database is an interactive, user-friendly, and comprehensive database that allows researchers to rapidly analyze gene and protein expression in various solid tumors [18]. We compared CYP27A1 expression level between primary tumor and normal using the UALCAN database. Furthermore, the diagnostic value of CYP27A1 in lung cancer was additionally assessed using the analysis platform TNMplot.com (www.tnmplot.com).

### Survival analysis for CYP27A1

LUAD patients were categorized into two subgroups based on the median value of CYP27A1 expression: high expression subgroup and low expression subgroup. Survival analysis was conducted by the Kaplan–Meier method and evaluated with the log-rank test. A prognostic classifier was constructed to assess whether CYP27A1 expression impacts the clinical outcomes of patients with LUAD. The prognostic value of CYP27A1 in lung cancer was further validated using Kaplan–Meier plotter.

### Investigation of CYP27A1 co-expression genes

LinkedOmics is a public website that contains multiple omics data for cancer research. We used the LinkFinder module of this database to identify the CYP27A1-related genes in LUAD. To assess the correlation between CYP27A1 and its co-expression genes, we employed the Pearson correlation coefficient. Furthermore, we utilized the LinkInterpreter module of the LinkedOmics platform to perform GSEA and derive the potentially pathways that may be mediated by CYP27A1 [19].

### Analysis of immune microenvironment

We utilized the R package GSVA to conduct ssGSEA analysis, comparing the proportions of immune cell infiltration between the CYP27A1-related subgroups. To evaluate the Stromal Score, Immune Score, and ESTIMATE Score, ESTIMATE software was used. Spearman correlation was used to analyze correlation the between the CYP27A1 expression and immune cell infiltrate level.

### Cell culture

The human normal bronchial epithelial cell line (16HBE) and lung cancer cell lines (A549, NCI-H1650, and NCI-H1299) were obtained from the Cell Bank of Chinese Academy of Sciences. These cells were cultured in RPMI-1640 medium, supplemented with 1% penicillin and streptomycin, and 10% fetal bovine serum (Gibco, China). The cultures were maintained at 37℃ in a 5% CO2 humidified atmosphere.

### Analysis of CYP27A1 expression level

RNA extraction from the cells was carried out using TRIzol reagent (Invitrogen, USA). Purified RNA (2 µg) was utilized for cDNA synthesis using a cDNA synthesis kit (Thermo Fisher, USA). Subsequently, qRT-PCR was conducted in a PCR System (Applied Biosystems, CA, USA). The CYP27A1 expression level was quantified using the 2^–ΔΔCt^ method. The primers can be found in Supplementary Table S2.

### Statistical analysis

The statistical analysis of data was conducted using the R software (version 3.3.3). The differences between tumor samples and adjacent noncancer samples were compared using the Wilcoxon signed-rank test. The receiver operating characteristic (ROC) analysis of CYP27A1 was performed using the pROC package of R. A *p*-value of less than 0.05 was considered statistically significant.

## Results

### Identifying prognosis-associated differentially expressed LRGs in LUAD patients

**Fig. 1:**
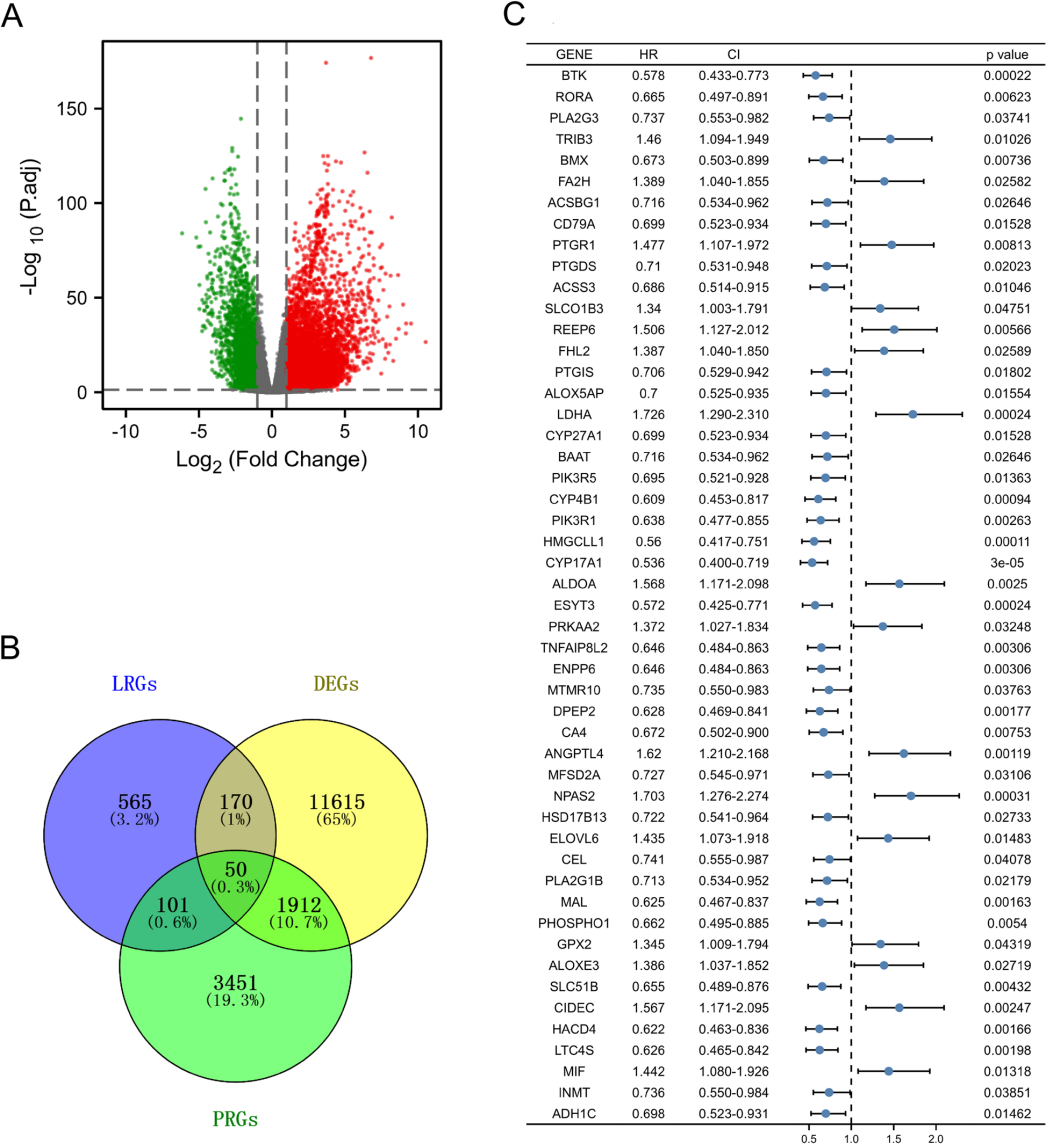
Identification of prognosis-associated differentially expressed LRGs in LUAD patients. **A** The volcano plots of DEGs. **B** The intersection genes of LRGs, DEGs, and PRGs. **C** The forest map of prognosis-associated differentially expressed LRGs

As presented in Fig. 1A a comprehensive count of 13747 DEGs was observed, with 10635 exhibiting up-regulation and 3112 showing down-regulation. Then, 50 prognosis-associated differentially expressed LRGs were obtained by the intersection of prognosis-related genes (PRGs), LRGs and DEGs (Fig. 1B). The univariate Cox regression analysis revealed a significant correlation between these prognostic genes and overall survival (Fig. 1C).

### The expression level of CYP27A1 in tumors

**Fig. 2:**
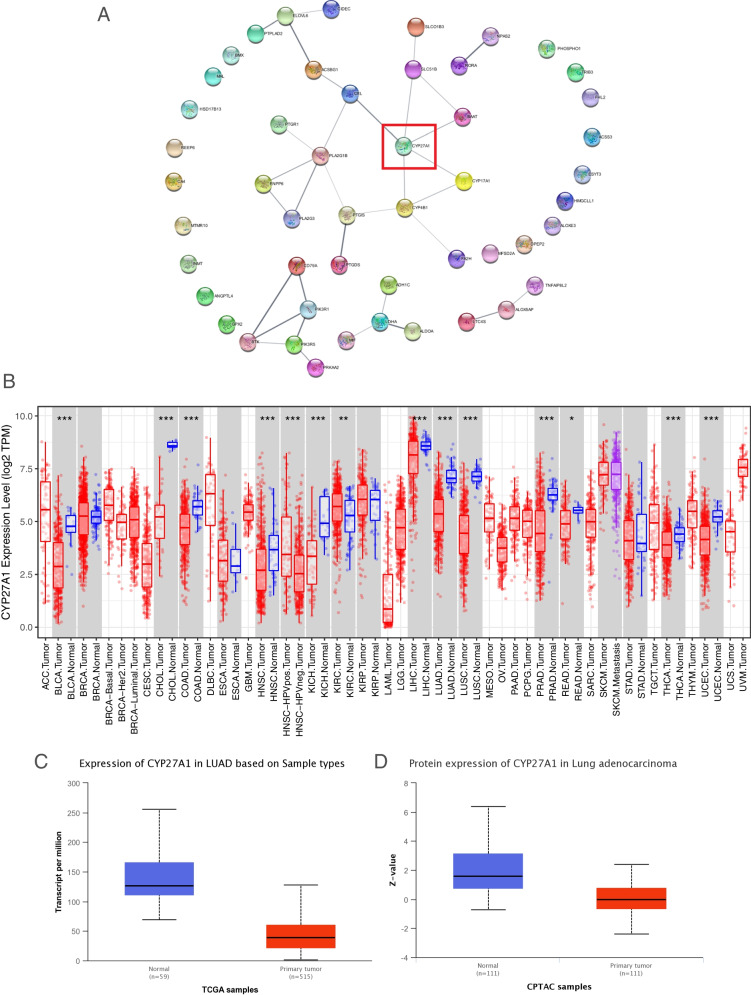
The expression level of CYP27A1 in tumors. **A** PPI network of 50 prognosis-associated differentially expressed LRGs. **B** CYP27A1 expression level in the pan cancers. **C** The mRNA expression of CYP27A1 in TCGA-LUAD dataset. **D** Protein expression of CYP27A1 in LUAD based on CPTAC samples. *p < 0.05, **p < 0.01, and ***p < 0.001

As shown in Fig. 2A, among these 50 LRGs, CYP27A1 has the highest degree. Therefore, we selected it for further analysis. Firstly, the CYP27A1 expression level in various tumors and normal samples was compared by TIMER data, and results indicated that CYP27A1 was decreased in bladder urothelial carcinoma (BLCA), cholangio carcinoma (CHOL), colon adenocarcinoma (COAD), head and neck squamous cell carcinoma (HNSC), kidney chromophobe (KICH), liver hepatocellular carcinoma (LIHC), LUAD, lung squamous cell carcinoma (LUSC), prostate adenocarcinoma (PRAD), rectum adenocarcinoma (READ), thyroid carcinoma (THCA), and uterine corpus endometrial carcinoma (UCEC) compared to normal samples. Compared to normal samples, CYP27A1 was up-regulated in kidney renal clear cell carcinoma (KIRC) (Fig. 2B). Besides, the UALCAN database was used to confirm the result. As shown in Fig. 2C-D, CYP27A1 gene and protein expression were decreased in primary tumor samples, as compared with these in the normal samples.

### Assessment of the correlation between CYP27A1 expression and the clinicopathological features of patients with LUAD

**Fig. 3:**
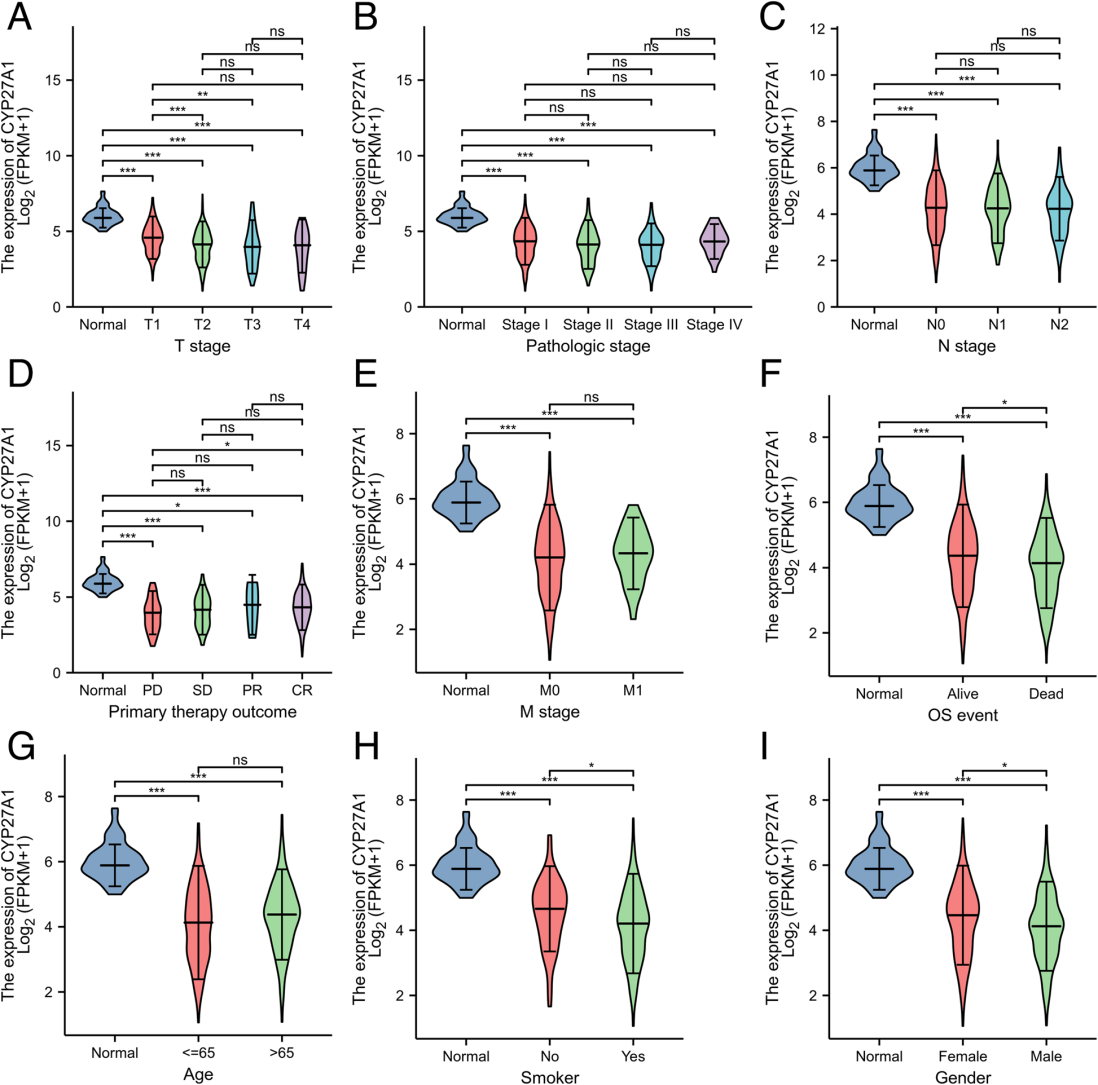
CYP27A1 expression in subgroups of TCGA-LUAD. Violin plots were presented for T stage (**A**), pathologic stage (**B**), N stage (**C**), primary therapy outcome (**D**), M stage (**E**), OS event (**F**), age (**G**), smoker (**H**), and gender (**I**). *p < 0.05, **p < 0.01, and ***p < 0.001

As presented in Fig. 3 and Table 1, we found the CYP27A1 expression level was down-regulated in T stage (T2/T3) (*p* < 0.01, Fig. 3A), primary therapy outcome (CR) (*p* < 0.05, Fig. 3D), OS event (dead) (*p* < 0.05, Fig. 3F), smoker (*p* < 0.05, Fig. 3H), and gender (male) (*p* < 0.05, Fig. 3I). However, the CYP27A1 expression showed no significant correlation with pathologic stage (Fig. 3B), N stage (Fig. 3C), M stage (Fig. 3E), and age (Fig. 3G).

**Table 1:** Cliniopathological parameters of low and high CYP27A1 expression group in TCGA-LUAD

Characteristic	Low expression of CYP27A1	High expression of CYP27A1	p
n	267	268	
T stage, n (%)			** < 0.001**
T1	63 (11.8%)	112 (21.1%)	
T2	161 (30.3%)	128 (24.1%)	
T3	32 (6%)	17 (3.2%)	
T4	11 (2.1%)	8 (1.5%)	
N stage, n (%)			0.995
N0	172 (33.1%)	176 (33.9%)	
N1	48 (9.2%)	47 (9.1%)	
N2	37 (7.1%)	37 (7.1%)	
N3	1 (0.2%)	1 (0.2%)	
M stage, n (%)			0.830
M0	189 (49%)	172 (44.6%)	
M1	12 (3.1%)	13 (3.4%)	
Pathologic stage, n (%)			0.179
Stage I	136 (25.8%)	158 (30%)	
Stage II	70 (13.3%)	53 (10.1%)	
Stage III	46 (8.7%)	38 (7.2%)	
Stage IV	12 (2.3%)	14 (2.7%)	
Primary therapy outcome, n (%)			0.106
PD	44 (9.9%)	27 (6.1%)	
SD	21 (4.7%)	16 (3.6%)	
PR	2 (0.4%)	4 (0.9%)	
CR	158 (35.4%)	174 (39%)	
Gender, n (%)			** < 0.001**
Female	121 (22.6%)	165 (30.8%)	
Male	146 (27.3%)	103 (19.3%)	
Age, n (%)			**0.008**
< = 65	143 (27.7%)	112 (21.7%)	
> 65	115 (22.3%)	146 (28.3%)	
Smoker, n (%)			**0.005**
No	26 (5%)	49 (9.4%)	
Yes	236 (45.3%)	210 (40.3%)	

### Decreased expression of CYP27A1 was associated with unfavorable prognosis in patients with LUAD

**Fig. 4:**
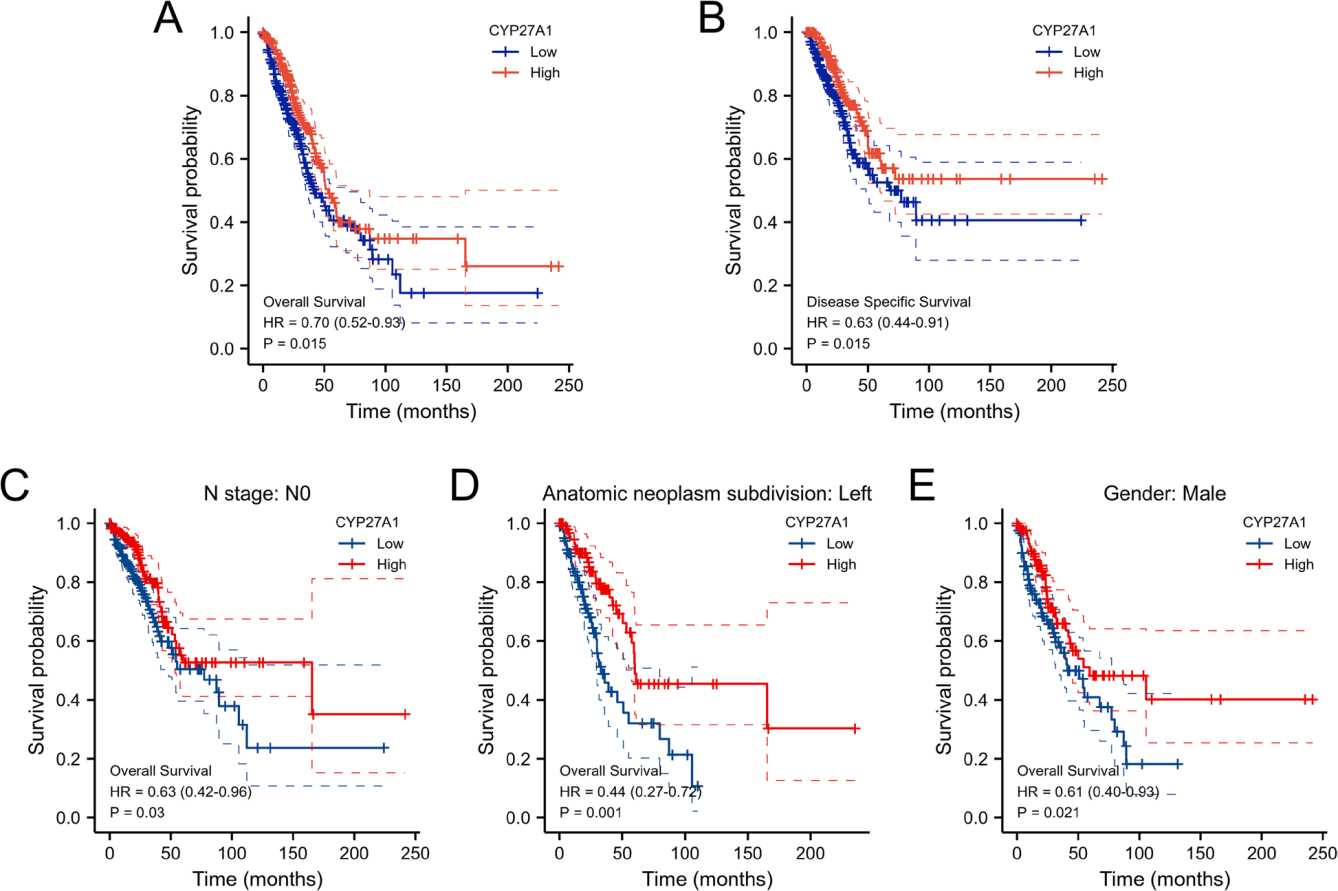
Prognostic value of CYP27A1 in the different subgroups. The overall survival (**A**) and disease-specific survival (**B**) in all LUAD patients. The overall survival in LUAD patients for N0 stage (**C**), anatomic neoplasm subdivision (**D**), and male (**E**) subgroups

Kaplan–Meier analysis showed that decreased expression of CYP27A1 was significantly related to poor overall survival (*p* = 0.015, Fig. 4A) and disease-specific survival (*p* = 0.015, Fig. 4B). Besides, the low CYP27A1 expression was also related to worse prognosis in the N0 subgroup of N stage (*p* = 0.03, Fig. 4C), left subgroup of anatomic neoplasm subdivision (*P* = 0.001, Fig. 4D), and male subgroup of gender (*p* = 0.021, Fig. 4E). Furthermore, univariate and multivariate Cox regression results indicated CYP27A1 was an independent prognostic marker for LUAD patients (Table 2). We also validated the prognostic value of CYP27A1 using an independent datasets (GSE41271 dataset, GSE11969 dataset, and Kaplan–Meier plotter), the results of which were consistent with those of the TCGA-LUAD dataset (Figure S1).

**Table 2:** Correlation analysis between CYP27A1 expression and OS analyzed by univariate and multivariate Cox regression

Characteristics	Total (N)	Univariate analysis		Multivariate analysis	
		Hazard ratio (95% CI)	*P* value	Hazard ratio (95% CI)	*P* value
T stage	501				
T1	168	Reference			
T2	269	1.454 (1.019–2.075)	**0.039**	1.460 (0.924–2.307)	0.105
T3&T4	64	3.030 (1.926–4.767)	** < 0.001**	2.387 (1.282–4.443)	**0.006**
N stage	492				
N0	325	Reference			
N1	94	2.387 (1.692–3.366)	** < 0.001**	1.492 (0.798–2.792)	0.210
N2&N3	73	2.974 (2.037–4.343)	** < 0.001**	1.266 (0.510–3.143)	0.611
M stage	360				
M0	335	Reference			
M1	25	2.111 (1.232–3.616)	**0.007**	2.446 (1.119–5.348)	**0.025**
Pathologic stage	496				
Stage I	270	Reference			
Stage II	119	2.469 (1.716–3.552)	** < 0.001**	1.427 (0.725–2.808)	0.303
Stage III	81	3.567 (2.438–5.218)	** < 0.001**	2.467 (0.895–6.798)	0.081
Stage IV	26	3.813 (2.197–6.618)	** < 0.001**		
Gender	504				
Female	270	Reference			
Male	234	1.060 (0.792–1.418)	0.694		
CYP17A1	504	0.178 (0.063–0.502)	**0.001**	0.213 (0.072–0.632)	**0.005**

### The diagnostic significance of CYP27A1

**Fig. 5:**
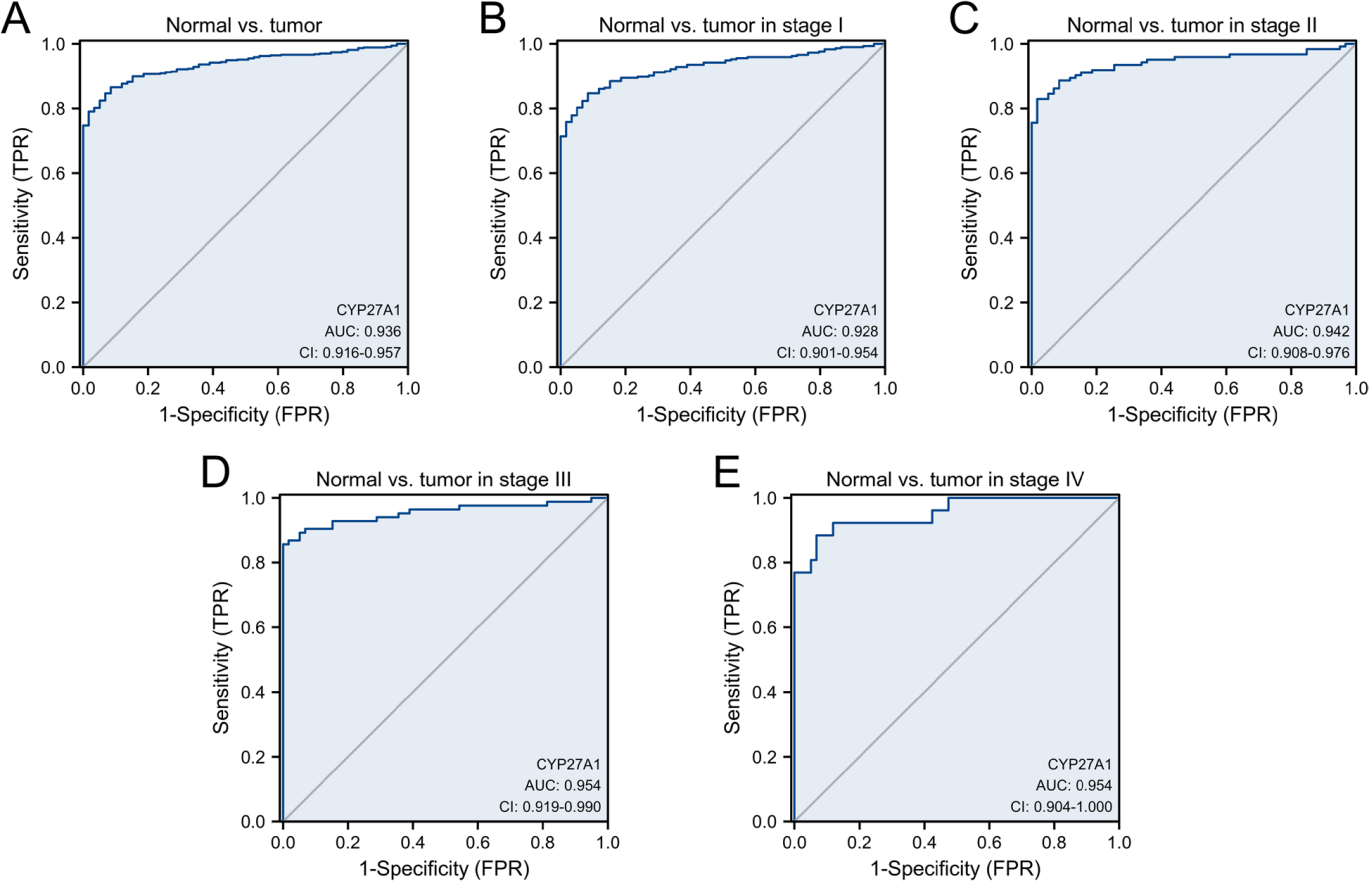
ROC curves of CYP27A1 expression in TCGA-LUAD (**A**), and stage I (**B**), II (**C**), III (**D**), and IV (**E**) subgroups

The ROC curves was applied to assess the diagnostic ability of CYP27A1, and the area under the curve (AUC) of CYP27A1 was 0.936 (Fig. 5A). Besides, we measured the CYP27A1 expression at stages I, II, III, and IV, the AUC value was 0.928 (Fig. 5B), 0.942 (Fig. 5C), and 0.954 (Fig. 5D), and 0.954 (Fig. 5E), respectively. We confirmed the diagnostic value of CYP27A1 using an independent datasets (TNMplot.com analysis platform, GSE11969 dataset, and GSE30219 dataset), the results of which were consistent with those of the TCGA-LUAD dataset (Figure S2).

### Analysis of the co-expression pattern of CYP27A1

**Fig. 6:**
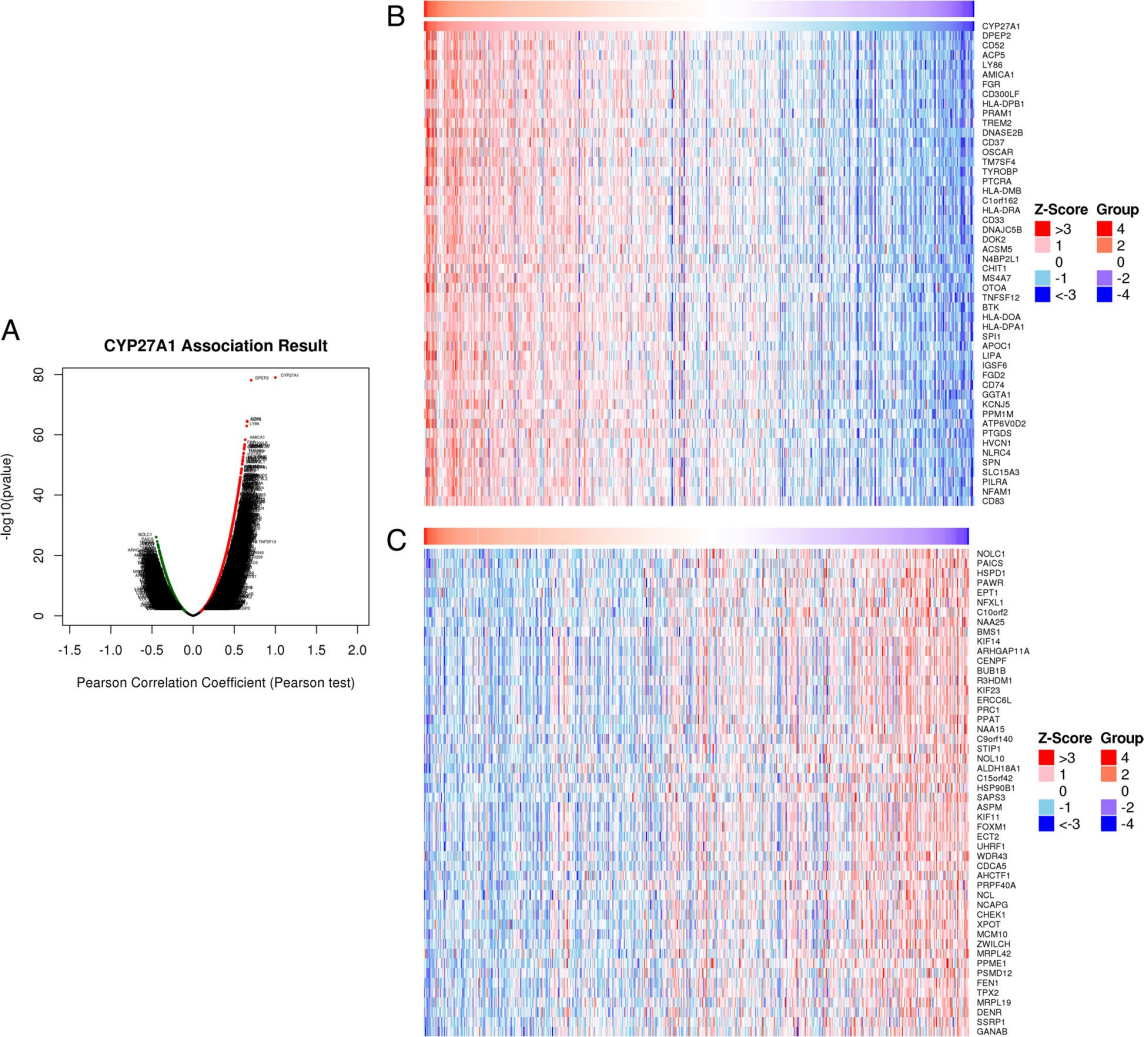
CYP27A1 co-expression genes in LUAD. **A** Volcano map of co-expressed genes of CYP27A1 based on the LinkedOmics database. **B** Top 50 genes with positive co-expression with CYP27A1 expression. **C** Top 50 genes with negative co-expression with CYP27A1 expression

**Fig. 7:**
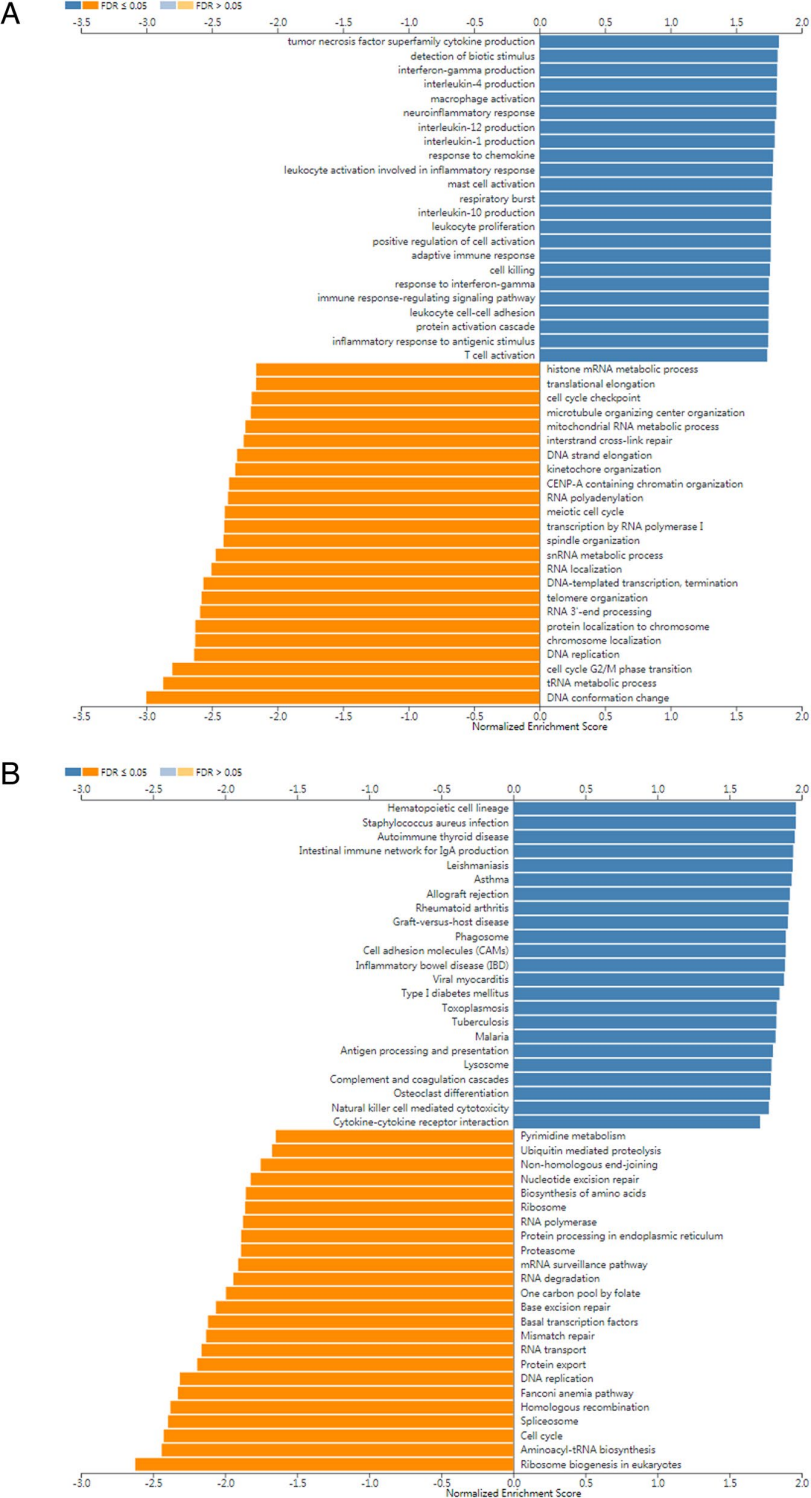
Immune- and inflammation-associated pathways medicated by CYP27A1 in LUAD. **A** GSEA revealed the correlation of CYP27A1 expression with GO biological process (**A**) and KEGG (**B**) depending on the LinkedOmics database

According to Fig. 6A, the findings revealed that 4446 genes (green dots) exhibited a negative correlation with CYP27A1, whereas 7385 genes (red dots) demonstrated a positive correlation with CYP27A1. Additionally, we constructed heat map displaying the top 50 genes positively (Fig. 6B) and negatively (Fig. 6C) associated with CYP27A1. Furthermore, GO-BP results showed that these co-expressed genes of CYP27A1 associated with tumor necrosis factor superfamily cytokine production, interleukin-4 production, macrophage activation, neuroinflammatory response, response to chemokine, leukocyte activation involved in the inflammatory response, mast cell activation, adaptive immune response, immune response-regulating signaling pathway, and T cell activation, etc. (Fig. 7A). KEGG results indicated that co-expressed genes of CYP27A1 significantly enriched in the intestinal immune network for IgA production, rheumatoid arthritis, cell adhesion molecules, inflammatory bowel disease, complement and coagulation cascades, natural killer cell-mediated cytotoxicity, cytokine-cytokine receptor interaction, etc. (Fig. 7B).

### CYP27A1 showed a correlation with the infiltration of the majority of immune cells

**Fig. 8:**
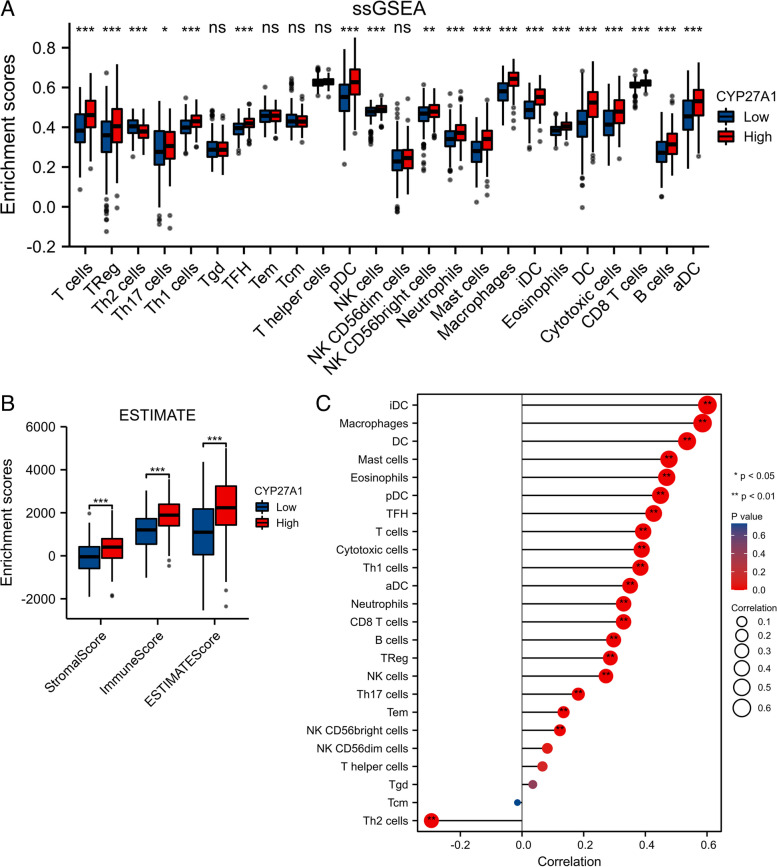
Immune cell infiltrates analysis of CYP27A1 in TCGA-LUAD. **A** The histogram presents the infiltration of immune cells between the low-CYP27A1 subgroup and high-CYP27A1 subgroup. **B** The histogram presents the Stromal score, Immune score, and ESTIMATE score between low-CYP27A1 subgroup and high-CYP27A1 subgroup. **C** Correlation analysis between 24 immune cell types level and CYP27A1 expression in LUAD

We used ssGESA to analyze the infiltration of immune cells, and we observed that aDC, B cells, CD8 T cells, cytotoxic cells, DC, eosinophils, iDC, macrophages, mast cells, neutrophils, NK CD56bright cells, NK cells, T cells, Treg, Th17 cells, Th1 cells, TFH, and pDC were increased, whereas Th2 cells were decreased in high-CYP27A1 subgroup compared to the low-CYP27A1 subgroup (Fig. 8A). ESTIMATE analysis showed that the Stromal score, Immune score, and ESTIMATE score were increased in the high-CYP27A1 subgroup compared with the low-CYP27A1 subgroup (Fig. 8B).

**Fig. 9:**
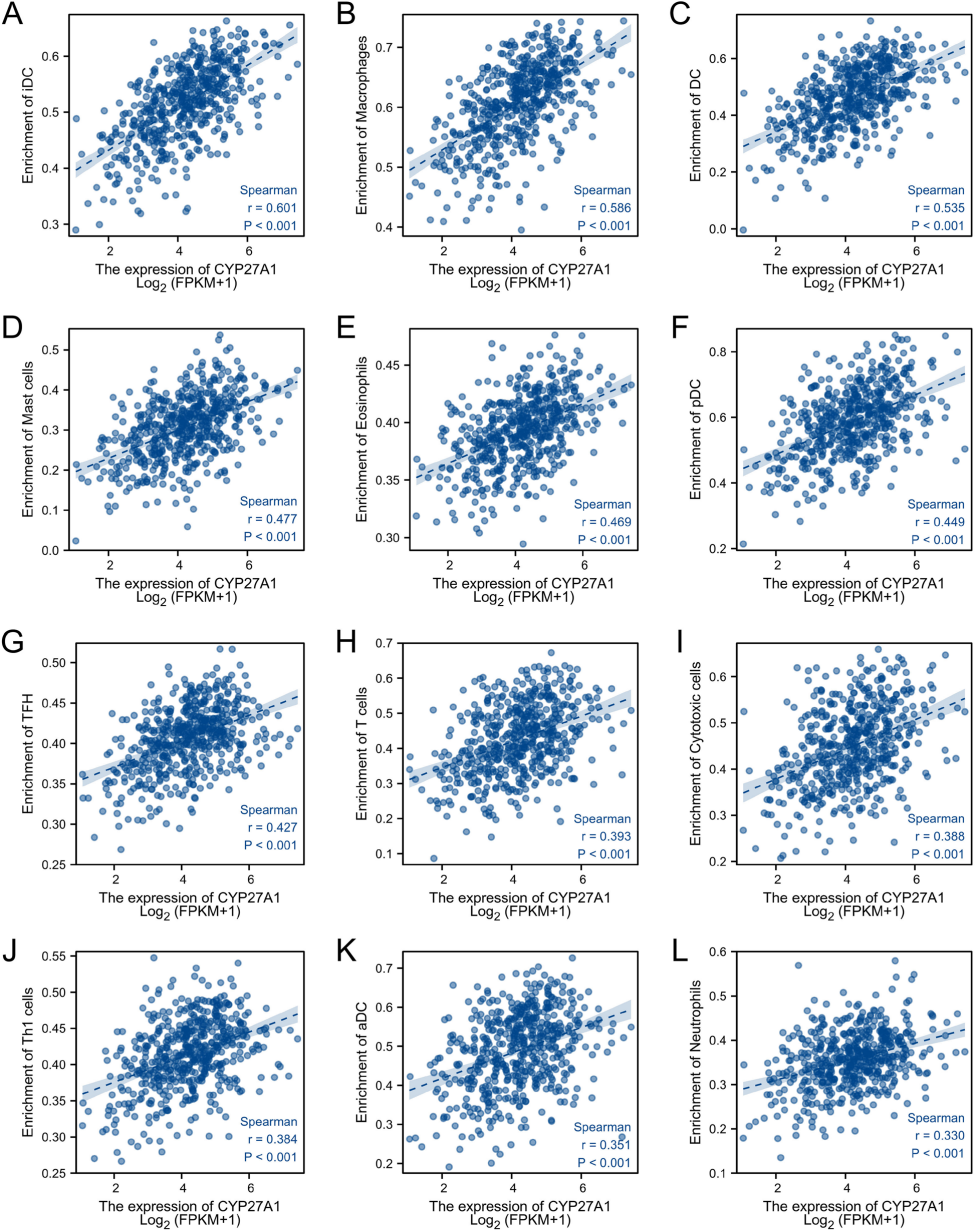
CYP27A1 expression positively correlated with iDC (**A**), macrophages (**B**), DC (**C**), mast cells (**D**), eosinophils (**E**), pDC (**F**), TFH (**G**), T cells (**H**), cytotoxic cells (**I**), Th1 cells (**J**), DC (**K**), and neutrophils (**L**) in LUAD

As shown in Fig. 8C, we observed a positive correlation between CYP27A1 expression and the majority of immune cell types, including iDC (*p* < 0.01, *r* = 0.60), macrophages (*p* < 0.01, *r* = 0.58), DC (*p* < 0.01, *r* = 0.53), mast cells (*p* < 0.01, *r* = 0.47), eosinophils (*p* < 0.01, 0.46), pDC (*p* < 0.01, *r* = 0.44), TFH (*p* < 0.01, *r* = 0.42), T cells (*p* < 0.01, *r* = 0.39), cytotoxic cells (*p* < 0.01, *r* = 0.38), Th1 cells (*p* < 0.01, *r* = 0.38), aDC (*p* < 0.01, *r* = 0.35), neutrophils (*p* < 0.01, *r* = 0.32), CD8 T cells (*p* < 0.01, *r* = 0.32), B cells (*p* < 0.01, *r* = 0.29), TReg (*p* < 0.01, *r* = 0.28), NK cells (*p* < 0.01, *r* = 0.27), Th17 cells (*p* < 0.01, *r* = 0.18), Tem (*p* < 0.01, *r* = 0.13), and NK CD56bright cells (*p* < 0.01, *r* = 0.12). CYP27A1 expression was negatively correlated with Th2 cells (*p* < 0.01, *r* = -0.29). Furthermore, the scatter plot also exhibited the correlation between CYP27A1 expression and various immune cell types (Fig. 9). In addition, we used an independent dataset (GSE11969 dataset) to validate the correlation between CYP27A1 expression and immune cells, and the results were largely consistent with those of the TCGA-LUAD dataset (Figure S3).

### Evaluation of the prognostic significance of CYP27A1 in relation to specific subgroups of tumor-infiltrating immune cells

**Fig. 10:**
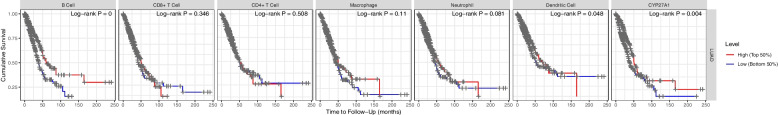
Survival curves of CYP27A1 in LUAD based on B cell, CD8 T cell, CD4 T cell, macrophage, neutrophil, and dendritic cell

Based on the above results, we speculated that CYP27A1 expression may impact prognosis due to tumor-infiltrating immune cells. Therefore, the TIMER database was applied to perform the prognostic assessment based on the CYP27A1 expression in immune cell subgroups. Our findings indicated that a reduced expression of CYP27A1 in B cell and dendritic cell subgroups was correlated with a worse prognosis (*p* < 0.05), whereas no significant difference was observed in the CD8 T cell, CD4 T cell, macrophage, and neutrophil subgroups (Fig. 10).

### qRT-PCR analysis of CYP27A1

**Fig. 11:**
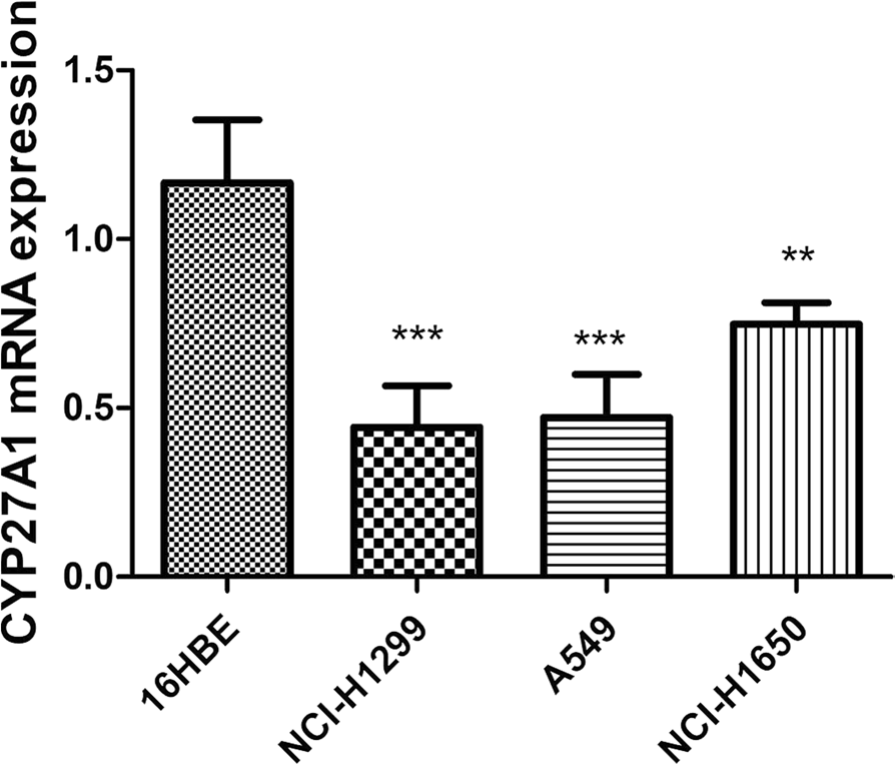
CYP27A1 expression in lung cancer cell lines (NCI-H1299, NCI-H1650 and A549) and 16HBE. **p < 0.01 and ***p < 0.001

We observed that CYP27A1 expression levels were downregulated in lung cancer cells (A549, NCI-H1650, and NCI-H1299) compared with 16HBE cells (*p* < 0.001) (Fig. 11).

## Discussion

LUAD has been considered a malignant tumor for its poor prognosis, refractory features, high mortality, and high incidence [2, 20]. It is crucial to identify potential biomarkers that facilitate cancer development and result in an unfavorable prognosis. This is essential for the development of potential therapies and the enhancement of patient care. Metabolic disorders have been demonstrated to affect tumor treatment response, invasion, proliferation, and growth. Thus, it has a significant impact on diverse malignancies [21]. Reprogramming of lipid metabolism is related to signal transduction, membrane synthesis, and energy production, thus affecting the drug resistance, immunity, and tumor microenvironment [22, 23]. Besides, the involvement of disrupted lipid metabolism in the initiation and progression of pulmonary tumors has been unveiled [24]. Based on the above reports, the regulation of lipid metabolism significantly impacts the onset and progression of lung cancer. However, its role is not completely understood in LUAD. Thus, it is crucial to discover potential biomarkers linked to lipid metabolism in LUAD. In this study, we have made a significant discovery by identifying 220 differentially expressed LRGs in patients with LUAD, which aligns with the impact of lipid metabolism on lung cancer development [24–26]. These findings strongly suggest the significant role of lipid metabolism in the development of LUAD, ultimately influencing patient outcomes. Furthermore, a recent study conducted single-cell RNA sequencing on various early-stage lung cancers, revealing a widespread dysregulation of lipid metabolism across different cell types [27]. This finding served as a catalyst for our investigation into genes associated with lipid metabolism. In the present study, a univariate Cox regression analysis revealed that 50 LRGs were significantly associated with overall survival. Among these genes, CYP27A1 has the highest degree and was selected for further analysis. We systematically assessed the expression level and clinical significance of CYP27A1 in LUAD. Based on our findings, a poor prognosis for LUAD patients was found to be associated with low expression of CYP27A1, which is consistent with the effect of CYP27A1 on prognosis in breast cancer [28].

CYP27A1, known as sterol 27-hydroxylase, is an enzyme belonging to the cytochrome P450 oxidase family and is predominantly expressed in liver tissue. It mainly catalyzes the hydroxylation step, regulates vitamin D3 metabolism, and sustains cellular cholesterol homeostasis [29, 30]. Notably, down-regulated CYP27A1 expression leads to lots of pathological processes related to bile acid and cholesterol metabolism. CYP27A1 was identified as a core LRGs in intervertebral disc degeneration [31]. It has been demonstrated that CYP27A1 exerts a significant influence in cholesterol metabolism in intestine cells [32]. Besides, the expression of CYP27A1 was found to be related to the proliferation of tumor cells, including those in colon, breast, and prostate cancer [33, 34]. CYP27A1 was reported to prevent bladder cancer cell proliferation via regulation of cholesterol metabolism [35]. CYP27A1 has been identified as a potential biomarker, and decreased expression of CYP27A1 has been linked to a worse prognosis in prostate cancer [36, 37]. It displayed distinct expression patterns in breast cancer, and a lower expression of CYP27A1 was found to be associated with a shorter overall survival [38], which was consistent with our findings. Further KEGG and GO-BP analyses indicated that CYP27A1 co-expression genes were associated with multiple signaling pathways, especially those associated with immune-related pathways, such as macrophage activation, leukocyte activation involved in the inflammatory response, mast cell activation, etc. These findings showed that CYP27A1 may exert complex regulation function in immune-related pathways.

The tumor microenvironment is composed of immune cells, endothelial cells, and stromal cells, and it can provide insights into the effectiveness of immunotherapy and patients' prognoses [39, 40]. Accumulating evidence has provided a clearer understanding that the tumor microenvironment exerts its influence on cancer prognosis through various pathways. For example, new findings indicate that the diversity in the hierarchical malfunction of T-cell exhaustion can have an effect on the prognosis of cancer, offering the potential to utilize it as a reliable predictor of outcomes in cancer patients [41]. The newly developed and robust tumor microenvironment-related risk model had significant implications for breast cancer patients in terms of overall survival [42]. Tumor-infiltrating immune cells are vital components of the tumor microenvironment, actively contributing to both the response to tumor therapy and the progression of tumors [43]. Therefore, we performed tumor-infiltrating immune cells analysis to gain a deeper understanding of the role played by CYP27A1 in LUAD. In the present study, our results demonstrated a significant positive correlation between the expression of CYP27A1 and most of immune cell infiltration. Additionally, elevated CYP27A1 expression has the potential to enhance antitumor immunity by attracting macrophages, CD8 T cells, B cells, NK cells, and Treg cells into the tumor microenvironment. These findings suggest that increased expression of CYP27A1 may enhance immune cell infiltration, which is linked to a positive prognosis in LUAD. This is in line with the conclusions drawn from other studies: the metastasis and growth of lung cancer are strongly influenced by the presence and activity of tumor-associated macrophages [44]; Th2 cells, mast cells, and TFH were related to the prognosis of early-stage of LUAD [45]; decreased B cell count was associated with a harmful prognosis in LUAD patients [46]. The above findings revealed the hypothetical function of CYP27A1 in regulating tumor-infiltrating immune cells.

Despite suggesting that CYP27A1 may serve as a valuable prognostic biomarker for LUAD patients, it is important to note its inherent limitations. Notably, all survival analysis data relied on public datasets, necessitating the need for validation in additional clinical cohorts. In addition, sufficient clinical samples need to be collected to validate CYP27A1 expression.

## Conclusion

In summary, our study suggested that low CYP27A1 expression is linked to a negative prognosis in LUAD patients, and it is also associated with immune infiltration levels in LUAD. Consequently, CYP27A1 has the potential to serve as a prognostic marker for LUAD patients, providing a foundation for further investigation into its role in the development and progression of lung cancer.

### Supplementary Information

**Additional file 1: Table S1.** The list of LRGs.

**Additional file 2:** **Table S2.** Sequences of primer used quantitative real-time PCR. **Figure S1.** Validation of the prognostic value of CYP27A1 in independent datasets. Prognostic analysis of CYP27A1 in GSE41271 dataset (A), GSE11969 dataset (B), and Kaplan-Meier plotter (C). **Figure S2.** Validation of the diagnostic value of CYP27A1 in independent datasets. ROC curves of CYP27A1 in TNMplot.com analysis platform (A), GSE11969 dataset (B), and GSE30219 dataset (C). **Figure S3.** Immune cell infiltrates analysis of CYP27A1 in GSE11969 dataset. (A) The histogram presents the proportion of 24 immune cell types between the CYP27A1 low expression group and CYP27A1 high expression group. (B) The histogram presents the Stromal sore, Immune score, and ESTIMATE score between the CYP27A1 low expression group and CYP27A1 high expression group. (C) Correlation analysis between 24 immune cell types level and CYP27A1 expression in GSE11969 dataset.

## Data Availability

All datasets used in this study are accessible in the TCGA (https://portal.gdc.cancer.gov/), UALCAN (http://ualcan.path.uab.edu/) databases, GSE41271 (https://www.ncbi.nlm.nih.gov/geo/geo2r/?acc=GSE41271), GSE11969 (https://www.ncbi.nlm.nih.gov/geo/geo2r/?acc=GSE11969), and GSE30219 (https://www.ncbi.nlm.nih.gov/geo/geo2r/?acc=GSE30219). Raw data are that verify the findings of this study are available on request from the corresponding author.
