# Supplementary material for: Transcriptomic analysis identifies CYP27A1 as a diagnostic marker for the prognosis and immunity in lung adenocarcinoma
Source: BMC Immunol. 2023 Oct 10;24:37. doi: 10.1186/s12865-023-00572-1 (PMC10565965; doi:10.1186/s12865-023-00572-1)
Supplement: Supplementary file 2 — Additional file 2: Table S2. Sequences of primer used quantitative real-time PCR. Figure S1. Validation of the prognostic value of CYP27A1 in independent datasets. Prognostic analysis of CYP27A1 in GSE41271 dataset (A), GSE11969 dataset (B), and Kaplan-Meier plotter (C). Figure S2. Validation of the diagnostic value of CYP27A1 in independent datasets. ROC curves of CYP27A1 in TNMplot.com analysis platform (A), GSE11969 dataset (B), and GSE30219 dataset (C). Figure S3. Immune cell infiltrates analysis of CYP27A1 in GSE11969 dataset. (A) The histogram presents the proportion of 24 immune cell types between the CYP27A1 low expression group and CYP27A1 high expression group. (B) The histogram presents the Stromal sore, Immune score, and ESTIMATE score between the CYP27A1 low expression group and CYP27A1 high expression group. (C) Correlation analysis between 24 immune cell types level and CYP27A1 expression in GSE11969 dataset. [file 12865_2023_572_MOESM2_ESM.docx]

**Table S2.** Sequences of primer used quantitative real-time PCR.

| Gene | Forward primer (5' to 3') | Reverse primer (5' to 3') |
| --- | --- | --- |
| CYP27A1 | AGCTGCGCTTCTTCTTTCAG | GCTCCATGTCGTTCCGTACT |
| GAPDH | TGAAGGTCGGAGTCAACGG | TGGAAGATGGTGATGGGAT |

Supplementary figures


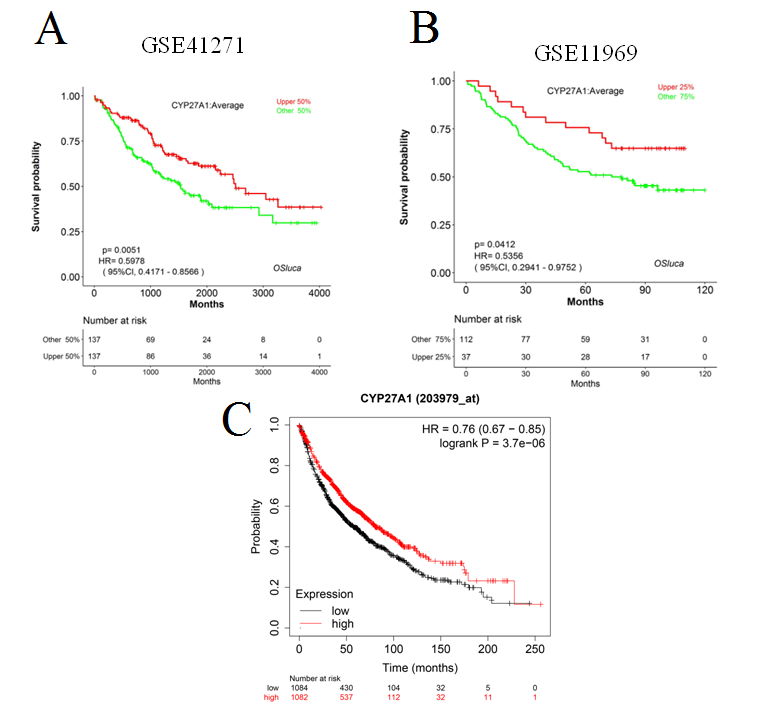


Figure S1 Validation of the prognostic value of CYP27A1 in independent datasets. Prognostic analysis of CYP27A1 in GSE41271 dataset (A), GSE11969 dataset (B), and Kaplan-Meier plotter (C).


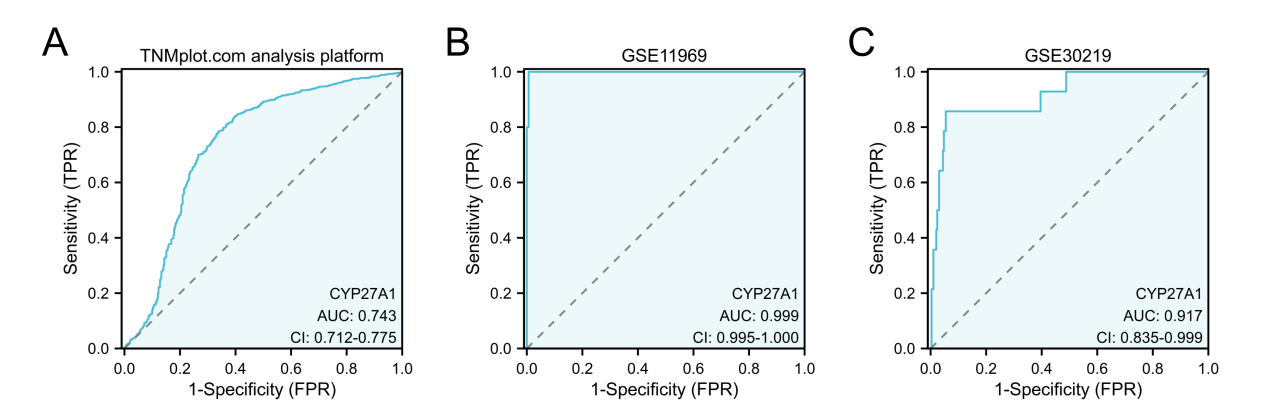


Figure S2 Validation of the diagnostic value of CYP27A1 in independent datasets. ROC curves of CYP27A1 in TNMplot.com analysis platform (A), GSE11969 dataset (B), and GSE30219 dataset (C).


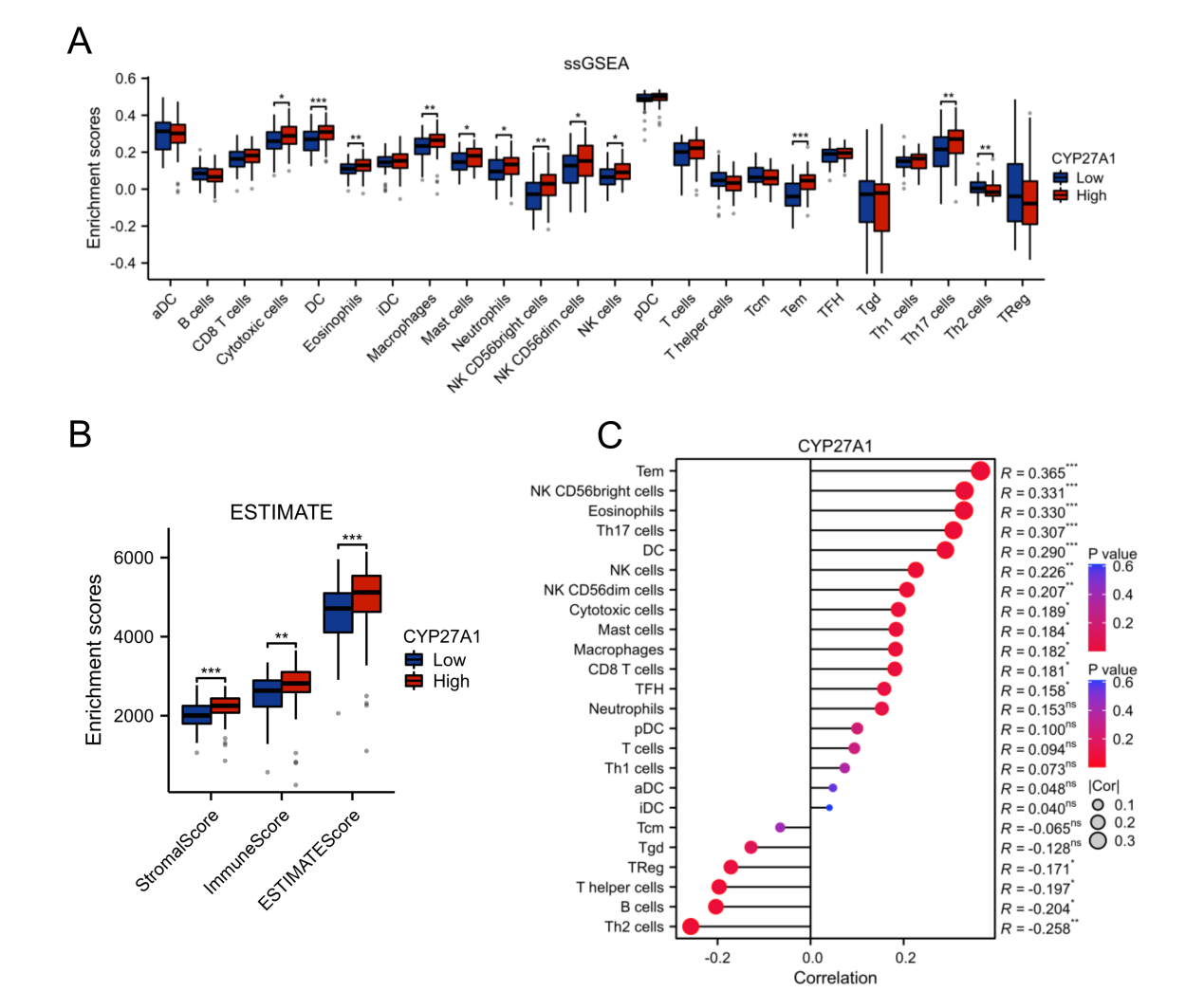


Figure S3 Immune cell infiltrates analysis of CYP27A1 in GSE11969 dataset. (A) The histogram presents the proportion of 24 immune cell types between the CYP27A1 low expression group and CYP27A1 high expression group. (B) The histogram presents the Stromal sore, Immune score, and ESTIMATE score between the CYP27A1 low expression group and CYP27A1 high expression group. (C) Correlation analysis between 24 immune cell types level and CYP27A1 expression in GSE11969 dataset.
